# Why do patients decline amniocentesis? Analysis of factors influencing the decision to refuse invasive prenatal testing

**DOI:** 10.1186/s12884-018-1812-3

**Published:** 2018-05-16

**Authors:** Pawel Sadlecki, Marek Grabiec, Pawel Walentowicz, Malgorzata Walentowicz-Sadlecka

**Affiliations:** Department of Obstetrics and Gynecology, Collegium Medicum in Bydgoszcz, Nicolaus Copernicus University of Torun, ul. Ujejskiego 75, 85-168 Bydgoszcz, Poland

**Keywords:** Amniocetesis, Decision making, Trisomy 21, Combined test

## Abstract

**Background:**

In recent years, determination of personalized risk for fetal chromosomal anomalies emerged as an important component of prenatal genetic counseling. Women in whom fetal risk for chromosomal aberrations is elevated are offered further testing. The aim of this study was to identify factors that may influence the decision to refuse invasive prenatal testing aimed at determination of fetal karyotype in a group of patients at increased risk of trisomy 21.

**Methods:**

The analysis included 177 patients with singleton pregnancy, whose personalized risk score for trisomy 21 calculated on the basis of the combined test exceeded 1:300. Diagnostic amniocentesis was performed in 125 patients from this subset, since the remaining 52 women declined invasive prenatal testing. The following factors were analyzed as potential determinants of the decision to refuse amniocentesis: maternal age (≥35 years), gravidity, number of miscarriages in previous pregnancies, educational status, marital status, indications to prenatal testing, gestational age at the time of prenatal testing, personalized risk score for fetal chromosomal aberrations and nuchal translucency (NT) value.

**Results:**

A statistically significant relationship was found between the decision to refuse amniocentesis and the number of previous miscarriages, maternal educational level, NT values and personalized risk score for fetal chromosomal aberrations. Multivariate logistic regression analysis identified primary maternal education and history of more than two miscarriages as independent significant predictors of declining amniocentesis. Women with personalized risk scores for trisomy 21 greater than 1:100 opted out of invasive prenatal diagnosis significantly less often than the remaining participants.

**Conclusion:**

In conclusion, the key role of high quality and accuracy of non-invasive diagnostic tests conducted in the first trimester should be emphasized as personalized risk score for fetal chromosomal aberrations determined based on their results is pivotal for further management of pregnancy. Equally important is to provide the patients with an accurate and comprehensible information about potential benefits and risks of invasive testing.

## Background

Prenatal diagnosis of Down syndrome allows for informed decision-making with regard to pregnancy continuation or termination. A number of screening strategies for detection of prenatal trisomy 21 in the first and second trimester have been developed. In Polish pregnant women, the screening typically involves ultrasonographic measurement of nuchal translucency (NT) at 11–14 weeks of gestation, along with the determination of blood levels of free-β-human chorionic gonadotrophin (βhCG) and pregnancy-associated plasma protein-A (PAPP-A) [1]. Based on the results of these tests, patient age and medical history, the so-called personalized risk score for fetal chromosomal aberrations is calculated. In line with the recommendations of the Polish Gynecological Society, the likelihood of fetal chromosomal aberration was considered high whenever the personalized risk score calculated on the basis of the combined test exceeded 1:300 [1]. The score determined on the basis of fetal NT at 11–13^+6^weeks of gestation and maternal age provides approximately 75% sensitivity in detection of trisomy 21, and inclusion of PAPP-A level in the screening algorithm further increases its sensitivity, up to ca. 85–90%, with only 5% false positive rate [2]. If the results of the screening point to an increased risk of fetal chromosomal aberrations, invasive testing, such as amniocentesis or trophoblast biopsy, is offered to pregnant women to determine fetal karyotype. However, the invasive tests are associated with increased risk of pregnancy complications, including miscarriage [3]. The aim of this study was to identify factors that may influence the decision to refuse invasive prenatal testing aimed at determination of fetal karyotype in a group of patients at increased risk of trisomy 21.

## Methods

A total of 2251 patients have been tested for chromosomal aberrations and fetal defects at the Prenatal Genetic Unit, Department of Gynecology, Obstetrics and Oncologic Gynecology, Nicolaus Copernicus University, Collegium Medicum in Bydgoszcz in 2014. A total of 2002 combined tests were conducted to calculate personalized risk score for fetal chromosomal aberrations on the basis of ultrasonographic markers NT, nasal bone (NB) at 45–84 mm CRL, serum levels of β-hCG and PAPP-A, in line with the recommendations of the Fetal Medicine Foundation (FMF) and Polish Gynecological Society [1, 2]. Some eligible patients refused to participate in the first trimester screening carried out at our Department; unfortunately, we were unable to estimate the size of this subgroup. Patients’ data were extracted from their medical histories obtained by two physicians specialized in medical genetics, employees of our Department. Both of them were trained in prenatal testing, and conducted a 20-min interview with each patient participating in the screening. Two counseling sessions were scheduled, one prior to non-invasive screening, and another one after the results were available (usually after 2 weeks). During the second session, the patients were advised if the invasive screening was required or not. Total number of diagnostic amniocenteses performed in 2014 was 202; aside from increased risk of fetal anomalies, other indications to invasive prenatal testing included maternal age > 35 years, history of chromosomal aberration or fetal defect in previous pregnancy, and abnormal result of the triple screen test. Eventually, the analysis included 177 patients with singleton pregnancy, whose personalized risk score for trisomy 21 calculated on the basis of the combined test exceeded 1:300. Diagnostic amniocentesis was performed in 125 patients from this subset, since the remaining 52 women declined invasive prenatal testing. The following factors were analyzed as potential determinants of the decision to refuse amniocentesis: maternal age (≥35 years), gravidity, number of miscarriages in previous pregnancies, educational status, marital status, indications to prenatal testing, gestational age at the time of prenatal testing, personalized risk score for fetal chromosomal aberrations and NT value. The protocol of the study was approved by the Local Bioethics Committee at Collegium Medicum in Bydgoszcz, Nicolaus Copernicus University in Torun (decision no. KB 239/2011), and written informed consent was sought from all the study participants. Statistical analysis of the results was carried out with PQStat ver. 1.6 package. Association between declining amniocentesis and explanatory variables were verified with chi^test. Moreover, all explanatory variables were tested as potential predictors of declining invasive prenatal diagnosis with univariate and multivariate logistic regression models. The relationships were considered significant at *p* < 0.05 and highly significant at *p* < 0.01.

## Results

**Fig. 1:**
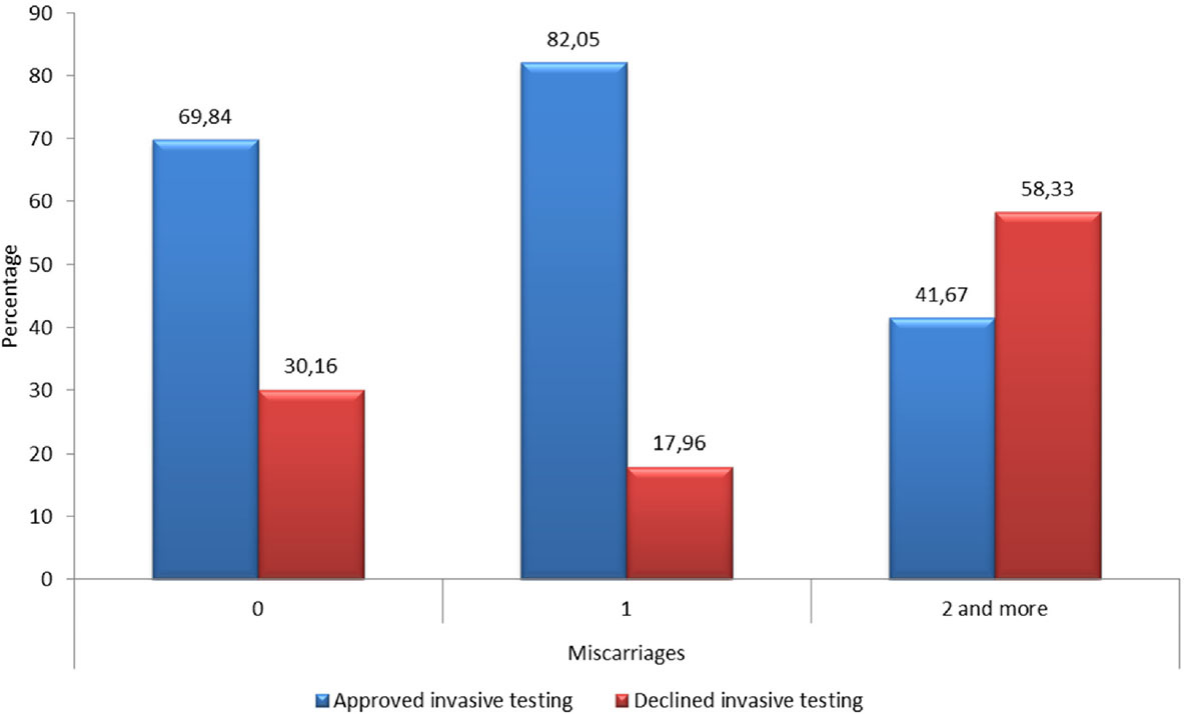
Distribution according to the number of miscarriages. Distribution of patients who declined amniocentesis according to the number of miscarriages in previous pregnancies (*p* = 0.0254)

A statistically significant relationship (chi^2 = 7.3417, df = 2, *p* = 0.0254) was found between the decision to refuse amniocentesis and the number of previous miscarriages. Women with a history of two or more miscarriages in previous pregnancies declined invasive prenatal testing significantly more often than the other patients (Fig. 1).

**Fig. 2:**
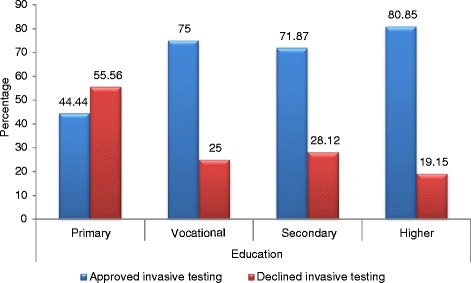
Distribution according to the educational level. Distribution of patients who declined amniocentesis according to their educational level (*p* = 0.0348)

The decision to refuse amniocentesis correlated significantly with maternal educational level (chi^2 = 8.6208, df = 3, *p* = 0.0348). Women with primary education declined invasive prenatal testing most often of all the study subjects (Fig. 2).

**Fig. 3:**
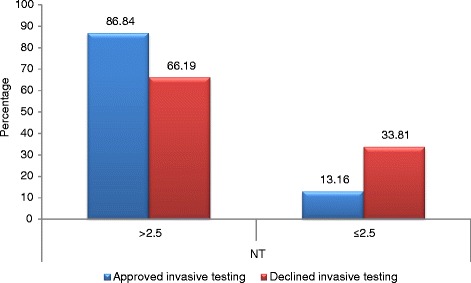
Distribution according to the nuchal translucency. Distribution of patients who declined amniocentesis according to nuchal translucency (NT) values (*p* = 0.0132)

A statistically significant relationship (chi^2 = 6.1364, df = 1, *p* = 0.0132) was observed between declining invasive prenatal diagnosis and nuchal translucency (NT) values. Patients whose fetuses presented with NT < 2.5 mm refused amniocentesis significantly more often than the other women (Fig. 3).

**Fig. 4:**
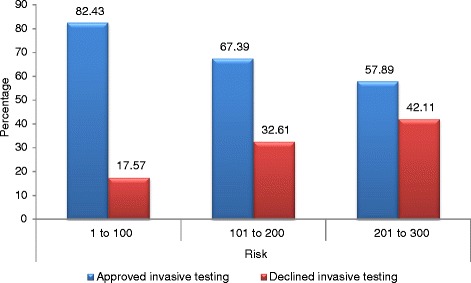
Distribution according to the personalized risk scores. Distribution of patients who declined amniocentesis according to their personalized risk scores for trisomy 21 (*p* = 0.0080)

A highly significant association (chi^2 = 9.6566, df = 2, *p* = 0.0080) was found between declining amniocentesis and personalized risk score for fetal chromosomal aberrations. The lower the personalized risk score for trisomy 21, the more often pregnant women refused prenatal invasive testing (Fig. 4).

The decision to refuse prenatal invasive testing did not correlate significantly with maternal age (< 35 years vs. ≥ 35 years; *p* = 0.94), gravidity (< 2 vs. ≥ 3; *p* = 0.99), marital status (married vs. others; *p* = 0.27) and gestational age at the time of the combined test (11 vs. 12 vs. 13; *p* = 0.42).

Logistic regression models to predict the decision to refuse amniocentesis on the basis of analyzed explanatory variables are presented in Table 1.

**Table 1:** Logistic regression models to predict the decision to refuse amniocentesis on the basis of analyzed explanatory variables

	b coefficient	*p*-value	Odds ratio	−95% CI	+ 95% CI
Intercept	− 0.894701	0.000525	0.40873	0.246506	0.677711
Primary education	1.312228	0.014799	3.714441	1.293011	10.6705
Miscarriages> 2	1.796904	0.025365	6.030948	1.248176	29.140396
Risk score > 1:100	−0.937951	0.027392	0.391429	0.170106	0.900716

Multivariate logistic regression analysis identified primary maternal education and history of more than two miscarriages as independent significant predictors of declining amniocentesis. Women with personalized risk scores for trisomy 21 greater than 1:100 opted out of invasive prenatal diagnosis significantly less often than the remaining participants.

## Discussion

The accuracy of prenatal cytogenetic diagnostic tests for fetal chromosomal anomalies has been confirmed over the last 40 years. Second trimester amniocentesis is the most commonly performed prenatal invasive diagnostic procedure. In recent years, determination of personalized risk for fetal chromosomal anomalies emerged as an important component of prenatal genetic counseling [4]. Women in whom fetal risk for chromosomal aberrations is elevated are offered further testing. However, the decision regarding invasive prenatal diagnosis needs to be made within tight timeframes since the window of gestational age during which such tests can be performed safely and provide accurate results is quite narrow [5]. Previous studies revealed that many women refer to prenatal screening and testing appointments with little understanding about the nature of the exams [5, 6]. Parents should understand the nature of the screening choices they have been offered; however, literature data imply that they may do not receive adequate, timely information from health professionals and thus are unable to make an informed decision [6]. The information and advice provided by an obstetrician prior to referral to a genetics center is likely an important determinant of maternal decision to undergo amniocentesis [7]. In our opinion, our study should be considered unique since it was conducted in Poland, whereas most similar previous studies were carried out in the United States or in Western Europe. To the best of our knowledge, the number of studies including subjects from Eastern Europe who refused invasive prenatal testing despite medical indications, is limited. We analyzed medical records of 177 patients qualified for amniocentesis, selected from a group of 2251 pregnant women tested for chromosomal aberrations and fetal defects. Nearly 30% of women from the study group refused invasive testing although their risk score for trisomy 21 exceeded 1:300; this fraction should be considered high. Other 125 patients underwent diagnostic amniocentesis. In the study conducted by Kuppermann et al., amniocentesis was carried out in 43 out of 710 women [8]. In our opinion, it is vitally important for pregnant women to understand the purpose and potential consequences of prenatal testing, especially that many patients seem to perceive non-invasive prenatal tests merely as a routine component of a larger laboratory workup [8, 9].

In our study, the decision to refuse amniocentesis was made most often by women with primary education; furthermore, primary education turned out to be the only independent predictor of declining invasive prenatal diagnosis on multivariate logistic regression analysis. However, this does not seem to be a universal rule since according to McCoyd, even relatively well-educated and economically stable persons may present with a surprising lack of knowledge and understanding on the purpose of the exam and possibility of fetal diagnosis [10]. During perinatal period, women are particularly vulnerable to anxiety and depressive disorders, and potential problems with their fetuses seem to contribute significantly to this conditions [11]. Patients in whom screening tests showed increased risk for fetal anomaly or revealed an actual defect, are typically devastated and confused [12]. The vast majority of pregnant women do not consider potential problems with their fetuses. Even upon referral to a medical appointment aimed at fetal health assessment, only a small proportion of patients discuss potential risk for a fetal anomaly with their partner or anyone else [9]. This implies that pregnant women display a relatively high level of denial or are just not prepared for possible diagnosis of fetal anomaly. This may explain why upon such diagnosis women are truly shocked and have a sense of betrayal, rather than being “only” surprised or saddened [8]. Pregnancy planning is just one indicator of preparedness for a gestation; the percentage of planned pregnancies in the United States is estimated at 50% [10]. Preparation for pregnancy can be considered a valuable resource for decision-making, especially in a crisis situation, such as detection of a fetal anomaly. Available evidence suggests that under such circumstances, women with unplanned pregnancies may be more prone to decision-making problems [13, 14]. The principal reasons behind undergoing prenatal tests are reassurance and the desire of knowledge about the fetal health. The decision to decline prenatal testing may be driven by personal views on pregnancy termination and the fear for iatrogenic pregnancy loss [13]. Although amniocentesis is associated with an increased risk for miscarriage, it should be stressed that the exact risk has still not been determined and its available estimates vary from 1:100 to 1:1600 [15]. In this study, women with a history of at least two miscarriages in previous pregnancies refused invasive prenatal diagnosis significantly more often than other subjects. Furthermore, the history of at least two miscarriages turned out to be an independent significant predictor of declining amniocentesis. The obstetric history of a woman is likely an important determinant of her decision to approve/refuse an invasive procedure [16]. Surprisingly, however, Sharda et al. showed that nearly 50% of women with unfavorable obstetric history and ultrasonographic evidence of soft aneuploidy markers agreed to take risk of amniocentesis rather than having an abnormal child. On the other hand, some women with two or more live children (including one with a genetic disorder) and primigravidas were reluctant to undergo an invasive procedure [16]. Still little is known on the factors that influence maternal decision to accept or decline diagnostic amniocentesis, and most of available data in this matter are not evidence-based or reflect solely personal experiences [17]. Potential determinants of the decision to undergo/refuse invasive prenatal testing include the method of conception, age, parity, consanguinity, family history of congenital anomalies, history of miscarriage, twin gestation, socioeconomic background and religion. In our series, declining amniocentesis did not correlate significantly with maternal age (< 35 years vs. ≥ 35 years), gravidity (< 2 vs. ≥3), marital status (married vs.others) and gestational age at the time of non-invasivescreening (11 vs. 12 vs. 13). Many women who conceived via assisted reproduction technology (ART) are older than 35 years, either due to previous unsuccessful attempts to get pregnant or as a consequence of conscious decision to delay childbearing [18]. Women who undergo in vitro fertilization (IVF) may theoretically be reluctant to diagnostic amniocentesis owing attributed risk of miscarriage. However, some of them may seek additional assurance that their pregnancies are karyotypically normal, owing past history of infertility. How these conflicting factors influencematernal decision to approve/decline prenatal testing is largely unknown [18]. Nevertheless, in our study, all pregnant women with a history of ART procedures and high personalized risk scores for trisomy 21 (*N* = 7) opted for an invasive testing. Also the patients whose fetuses presented with nuchal translucency values > 2.5 mm and those with personalized risk scores for trisomy 21 greater than 1:100 refused amniocentesis significantly less often than the remaining participants of this study. Patients who did not give their consent for invasive tests can be offered non-invasive prenatal testing (NIPT) [19, 20]. Rapid advances in NIPT based on cell-free fetal DNA in maternal plasma have now made it possible to identify pregnancies affected by trisomy 21 from 10 weeks of gestation; the blood test provides high accuracy (> 99%) and low false-positive rate (0.1%) in identification of affected pregnancies [21]. NIPT has two key clinical advantages over invasive testing: it does not pose a risk of miscarriage and can be conducted early in pregnancy. However, it should be remembered that NIPT is not currently considered fully diagnostic, and therefore, its positive result needs to be verified by means of an invasive testing [22]. Widespread use of fetal cell-free DNA testing would with no doubt reduce the number of unnecessary invasive tests (amniocentesis and chorionic villi sampling) and eliminate associated risk for miscarriage [23]. Moreover, the use of NIPT may exert a salient effect on pregnant woman’s general attitude to prenatal genetic testing. Many patients who would currently decline prenatal genetic testing would likely opt for performing NIPT [24].

## Conclusion

In conclusion, the key role of high quality and accuracy of non-invasive diagnostic tests conducted in the first trimester should be emphasized as personalized risk score for fetal chromosomal aberrations determined based on their results is pivotal for further management of pregnancy. Equally important is to provide the patients with an accurate and comprehensible information about potential benefits and risks of invasive testing. In our opinion, patients’ educational level may influence their decisions regarding testing, and thus, all pregnant women should be provided with a clear information about available prenatal diagnostic options and their outcomes. Furthermore, it should be remembered that a subset of patients who declined invasive prenatal testing still may give their consent for NIPT.

## Abbreviations

ART: Assisted reproduction technology
CRL: Crown rump length
FMF: Fetal Medicine Foundation
IVF: In vitro fertilization
NB: Nasal bone
NIPT: Non-invasive prenatal testing
NT: Nuchal translucency
PAPP-A: Pregnancy associated plasma protein - A
βhCG: Free β – human chorionic gonadotropin
